# Editorial: Understanding Lung Acinar Micromechanics in Health and Disease: Linking Quantitative Imaging and Organ Scale Mechanics by Computational Modeling

**DOI:** 10.3389/fphys.2020.640398

**Published:** 2021-01-20

**Authors:** Lars Knudsen, Matthias Ochs, Bradford J. Smith

**Affiliations:** ^1^Institute of Functional and Applied Anatomy, Hannover Medical School, Hannover, Germany; ^2^Biomedical Research in Endstage and Obstructive Lung Disease Hannover (BREATH), Member of the German Center for Lung Research (DZL), Hannover, Germany; ^3^Institute of Functional Anatomy, Charité—Universitaetsmedizin Berlin, Berlin, Germany; ^4^German Center for Lung Research (DZL), Berlin, Germany; ^5^Department of Bioengineering, College of Engineering, Design, and Computing, University of Colorado Denver | Anschutz Medical Campus, Aurora, CO, United States; ^6^Section of Pulmonary and Sleep Medicine, Department of Pediatrics, School of Medicine, University of Colorado, Aurora, CO, United States

**Keywords:** acinar micromechanics, intravital microscopy, computational modeling, stereology, surfactant, ventilation induced lung injury (VILI)

The respiration-related dynamic changes in acinar microarchitecture are very complex and not entirely understood. Therefore, the overall goal of this special issue was to present studies of quantitative morphology, lung mechanics, and computational simulations of acinar dynamics under both physiological and pathophysiological conditions. Taken together, this collection of articles includes four reviews, one hypothesis and theory article, and nine publications of original research.

Matuszak et al. summarize the history and technical developments in the field of intravital microscopy which provides two-dimensional images for assessments of dynamic changes of subpleural alveoli *in vivo*. Taking both intravital microscopy and other imaging techniques into account, the authors comprehensively describe the dynamic changes of both the alveolar airspaces and the capillary network within alveolar septa. This highlights the complex three-dimensional architecture of the alveolar capillary network which is subject to dynamic changes during respiration, and developmental related changes that have not been studied in detail before. In a new-born mouse lung Buchacker et al. used Serial Block-Face Scanning Electron Microscopy to create three-dimensional image stacks with nanometer-scale resolution and then segmented the capillary network within alveolar septa. The reconstruction also included feeding arterioles and the draining venules to describe the complete path of blood through this network. Of particular interest for physiology, and potential use for computational modeling, is the finding that the capillary networks of different saccules are highly interconnected and receive blood from several arterioles and, as such, cannot be regarded separately.

While Buchacker et al. were interested in the capillary network, Cremona et al. investigated the development and Schulte et al. effects of aging on the pulmonary airspaces with the goal of linking structure to function. Both of those studies used design-based stereology, the current gold standard for quantitative morphology using any imaging technique. Cremona et al. investigated the relevance of the integrin α8 subunit in lung development and found impaired prenatal branching morphogenesis of the conducting airways with reduced lung volume at birth. Postnatal alveolarization was characterized by accelerated formation of secondary septa so that at late time points lung volumes did not differ between wildtype and integrin α8 subunit-deficient mice (Cremona et al.). The effects of aging on lung structure and organ-scale lung mechanics in mice was further investigated by Schulte et al. Aging was linked with an increase in the volumes of alveolar and ductal airspaces, as well as alveolar number, while organ-scale tissue elastance decreased. These observations indicate an age-related structural remodeling that result in more distensible lungs.

The analytical and computational modeling of Bou Jawde et al. suggests that the age-related reductions in lung stiffness described by Schulte et al. arise from the alterations in alveolar size and alveolar number, not from changes in the properties of the septal tissue itself. By fitting their models to parenchymal stiffness measured in young and old mice, they show that the age-related stiffening of the alveolar septa may be attributed to an increase in stiffness of the load-bearing fibers in the alveolar septa (Bou Jawde et al.). In contrast to that highly detailed analysis of the intra-septal fibers and septal mechanics, Ma et al. present a detailed computational model of the whole human lung that links tissue and biofluid mechanics. Their approach combines different length scales including purely conductive airways that are embedded in parenchymal airspaces which are stabilized by the presence of a dynamic pulmonary surfactant system with realistic physicochemical dynamics. The resulting large system of equations was solved for the healthy human lung using high performance computing techniques and their modeling approach provides a cutting-edge tool for investigating lung dynamics on scales from the molecular to the whole organ.

Perlman presents a conceptual analysis of how increased surface tension affects septal wall mechanics based on imaging studies performed in an *ex-vivo* ventilated and perfused lung system to model different mechanism involved in acute lung injury. An edema filled alveolus suffering from high surface tension leads to a deformation of the intervening and radiating septa of adjacent alveoli. This resulting stress concentration is directly proportional to surface tension and is an important pathogenic force in ventilation-induced lung injury *via* alveolar interdependence. Perlman further discusses therapeutic interventions to reduce surface tension and why surfactant replacement therapy might not be effective in acute lung injury. While these concepts were developed and are most frequently discussed in the context of acute lung injury, Sehlmeyer et al. extensively reviewed the roles of two important components of surfactant, Surfactant Protein C (SP-C) and cholesterol, in chronic lung diseases such as pulmonary fibrosis. The authors explore the interactions between SP-C, cholesterol, and lung mechanics in healthy lungs and summarize the current understanding of how abnormalities in either of these components (e.g., SP-C deficiency) might result in a pathway of chronic impairment of alveolar dynamics, elevated mechanical stresses, chronic lung injury, and eventual fibrosis.

These factors play out on an acute timescale during ventilator-induced lung injury as detailed in two experimental studies that describe pathologic structural changes and organ-scale lung mechanics using animal models. Smith et al. subjected mice to injurious mechanical ventilation and investigated acinar microarchitecture at different airway opening pressures to infer abnormalities in alveolar dynamics that correspond with degraded surfactant function and lung mechanics. The authors identified three alveolar fates directly reflected in organ-scale lung mechanical abnormalities: (1) unrecruitable flooded alveoli; (2) unstable alveoli that cyclically recruit and derecruit; and (3) healthy and stable alveoli. Unrecruitable alveoli due to high surface tension (microatelectases) have also been show to occur in acute bleomycin-induced lung injury and might act as stress concentrators that potentially increase the risk for ventilation-induced lung injury. To investigate these stress concentrations, Albert et al. ventilated bleomycin pre-injured lungs to show that the presence of occult microatelectases was associated with the development of ventilator-induced lung injury as measured in increased blood-gas barrier permeability, interstitial edema and organ-scale tissue elastance (Albert et al.). These data illustrated that regional heterogeneity in lung ventilation due to derecruited alveoli increase the risk of lung injury during mechanical ventilation in rodents.

Herrmann et al. used four-dimensional computed tomography to quantify heterogeneity in regional ventilation and parenchymal tidal strain in a porcine model of acute lung injury. This large animal model allows consideration of the important gravitational effects such as tidal strain in the dependent lung. After injury induction, the authors observed that heterogeneity in ventilation and regional parenchymal deformation could be reduced by means of multi-frequency oscillatory ventilation (MFOV) compared to a conventional single-frequency ventilation protocol. Accordingly, MFOV is a promising lung-protective ventilation strategy for injured lungs due to more uniform stress distribution.

In acute- and ventilator-induced lung injury, derecruitment of lung parenchyma contributes to ventilation-induced lung injury *via* atelectrauma (repetitive tidal recruitment and derecruitment) and volutrauma (overdistension) in the remaining patent airspaces. In this context Mori et al. developed an analytical mathematical model of the dynamics of derecruitment of distal airspaces during the progression of ventilator-induced lung injury. Their model reproduces the time course of tissue elastance measurements by assigning time- and tidal volume-dependent distributions of opening and closing pressures, as well as opening and closing rates, to lung units. Hence, changes in lung elastance after recruitment maneuver can be described by a progressive time- and pressure-dependent derecruitment of airspaces.

The (patho)physiology of the alveoli in acute lung injury, or alveolar micromechanics, is summarized in the context of mechanical ventilation by Kollisch-Singule et al. taking into account imaging studies (e.g., *in vivo* microscopy) and computational simulations. The authors identify alveolar instability, alveolar size heterogeneity, alveolar interdependence, and alveolar tidal volumes as critical parameters during mechanical ventilation and describe how changes in the ventilation parameters influence alveolar micromechanics. The authors conclude that the choice of the optimal ventilation pattern for an injured lung should take the pathophysiology of the alveoli into account, above all how alterations in the ventilation protocol affect alveolar dynamics through the respiratory cycle. Based on these pathophysiological concepts of the alveolar micromechanics Nieman et al. discuss the challenges of the mechanical ventilation of patients suffering from acute respiratory distress syndrome (ARDS) and introduce different ventilation strategies and related clinical trials which might be able to minimize ventilation-induced lung injury.

We hope that this collection of articles stimulates further research in this exciting and clinically relevant field of pulmonary physiology which very much depends on inter-disciplinarity research.

## Author Contributions

LK, MO, and BS wrote, edited, and approved the final version of the Editorial. All authors contributed to the article and approved the submitted version.

## Conflict of Interest

The authors declare that the research was conducted in the absence of any commercial or financial relationships that could be construed as a potential conflict of interest.

